# Independent Walking as a Major Skill for the Development of Anticipatory Postural Control: Evidence from Adjustments to Predictable Perturbations

**DOI:** 10.1371/journal.pone.0056313

**Published:** 2013-02-07

**Authors:** Fabien Cignetti, Milan Zedka, Marianne Vaugoyeau, Christine Assaiante

**Affiliations:** 1 Laboratoire de Neurosciences Cognitives (LNC - UMR 7291), Centre National de la Recherche Scientifique, Marseille, France; 2 Aix-Marseille Université, Marseille, France; 3 Fédération de Recherche N3512, Comportement-Cerveau-Cognition, Marseille, France; 4 Division of Paediatric Neurology, Department of Paediatrics, Inselspital, University of Bern, Bern, Switzerland; Goldsmiths, University of London, United Kingdom

## Abstract

Although there is suggestive evidence that a link exists between independent walking and the ability to establish anticipatory strategy to stabilize posture, the extent to which this skill facilitates the development of anticipatory postural control remains largely unknown. Here, we examined the role of independent walking on the infants’ ability to anticipate predictable external perturbations. Non-walking infants, walking infants and adults were sitting on a platform that produced continuous rotation in the frontal plane. Surface electromyography (EMG) of neck and lower back muscles and the positions of markers located on the platform, the upper body and the head were recorded. Results from cross-correlation analysis between rectified and filtered EMGs and platform movement indicated that although muscle activation already occurred before platform movement in non-walking infants, only walking infants demonstrated an adult-like ability for anticipation. Moreover, results from further cross-correlation analysis between segmental angular displacement and platform movement together with measures of balance control at the end-points of rotation of the platform evidenced two sorts of behaviour. The adults behaved as a non-rigid non-inverted pendulum, rather stabilizing head in space, while both the walking and non-walking infants followed the platform, behaving as a rigid inverted pendulum. These results suggest that the acquisition of independent walking plays a role in the development of anticipatory postural control, likely improving the internal model for the sensorimotor control of posture. However, despite such improvement, integrating the dynamics of an external object, here the platform, within the model to maintain balance still remains challenging in infants.

## Introduction

The ability to control action prospectively over the first years of life, which involves having priori knowledge of actions effects, constitutes a cornerstone for action development [1], [2]. A powerful mean to examine this feature consists in studying developmental changes in postural control when performing a motor task. Postural control in fact supports motor action by ensuring balance during execution of action [3]. Then, it is necessary that postural adjustments anticipate for the imbalance produced by actions [4]–[6].

Several studies have been conducted on the development of postural anticipation when reaching in sitting and draw a twofold conclusion. First, anticipation in sitting postural control emerges as early as the first year of life, with 4- to 12-month-old infants demonstrating some ability to activate the postural (neck and trunk) muscles before the arm muscle that initiates the arm movement [7]–[10]. Second, postural anticipation to prepare for the reach becomes consistent only in late infancy, with an increased anticipatory activity of the postural muscles in infants aged over 15 months [10]. Such a developmental trend was further supported by findings from another study concerned with the development of anticipatory postural adjustments during a pulling task while standing [11]. The proportion of pulls involving anticipatory activation of leg muscle to prospectively counteract an expected forward displacement in the body’s center of gravity progressively increased between 10 and 17 months. Interestingly, regrouping data in terms of infants with and without independent walking experience revealed marked increases in the temporal specificity and consistency of anticipatory adjustments as infants gain experience with walking.

In the above framework, a study on the use of a contact surface for stabilizing upright posture provided further details on the link between independent walking and the development of anticipatory control [12]. It was demonstrated that infants with no or very little walking experience used contact surface in reaction to their body sway, such like a mechanical support stabilizing posture. Inversely, infants with significant walking experience (∼ 1.5 month post-walking) used it in anticipation to body sway, integrating somatosensory information before swaying to stabilize posture. Walking appeared consequently as a crucial piece for establishing anticipatory strategy to stabilize posture, likely due to the refinement of an internal model for the sensorimotor control of posture that starts occurring at the time infants walk. However, there is evidence that infants within the first months of independent walking demonstrate anticipatory lateral weight shifts before initiating gait, suggesting that some form of anticipatory postural control is already present at independent walking onset [13]–[15].

In sum, although postural anticipation is likely already in use before infants start walking, the onset of walking experience seems to bring about significant changes in anticipatory postural control. The purpose of this study, therefore, was to investigate whether independent walking experience plays a facilitative role in the development of anticipatory postural control. To this end, we evaluated whether walking infants show greater sophistication compared to non-walking infants in their adult-like ability to anticipate for postural disturbances induced by a continuously moving platform. Indeed, there is evidence in adult population that anticipatory postural adjustments prevail when the platform moves in a rhythmic fashion, involving the activation of appropriate muscles prior to the turning points of the platform to counteract the destabilization [16]–[19]. This comes from the fact that the postural challenge induced by the moving platform is highly predictable and the ongoing sensory inputs can be used by the central nervous system to foretell the mechanical effect of the perturbation. Specifically, the experiment included both infants (some of whom were independent walkers) and adults, who were sitting on a support surface continuously rotating in the frontal plane that caused postural destabilization (i.e., lateral tilt). The subjects’ ability to anticipate for the destabilization induced by the moving platform was examined from the temporal relationship between the muscular activation of neck (i.e., trapezius muscle) and back (i.e., erector spinae muscle) muscles, which function is to counteract lateral postural sway. We predicted that only infants walking independently would activate these muscles in anticipation of the platform rotations to counteract the forthcoming destabilization as adults do. Moreover, we also examined the kinematic behaviour of the subjects to reveal the strategies used to cope with the balance challenge.

## Methods

### Subjects

Subjects were 10 infants who had not attained independent walking yet (aged 6–13 months, 5 boys), 10 infants who were independent walkers (aged 10–22 months, 7 boys) and 6 young adults (aged 18–30 years, 3 men). The more mature infants within the group of infants at the pre-walking stage were able to stand upright (1 girl, 3 boys) while the less mature ones were still mastering upright posture. Independent walkers had one to six months of walking experience, determined as the time interval from walking onset (i.e., the infant was able to perform at least three independent walking steps) until the testing day. Walking onsets were obtained through parental reports. All subjects were recruited from the community of Marseille and were in the majority from middle-class families. The infants’ caregivers and the young adults gave written informed consent. The local research ethics committee CPP Sud-Méditerranée I approved the experiment.

### Apparatus

**Figure 1:**
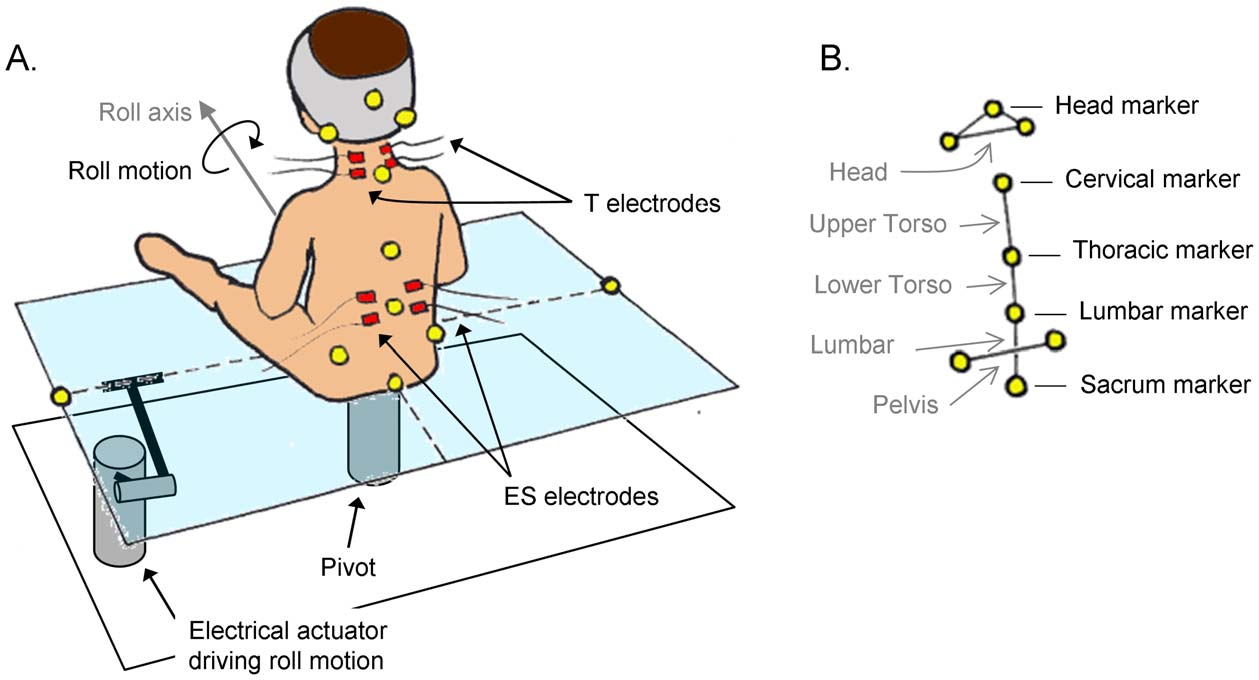
The experimental set-up. (A) The participant sat on a platform that produced continuous roll rotations. Surface electrodes were placed over erector spinae (i.e., ES) and trapezius (i.e., T) muscles. Reflective markers were placed on anatomical landmarks of the upper body and the head and on the platform. (B) Body segments were defined based on the markers’ locations.

The subject sat on a software-controlled, electrically driven rotating platform (90 cm×90 cm) that produced sinusoidal roll rotations at a frequency of 0.5 Hz (Fig. 1A). The peak-to-peak platform rotation was 6.6° and the duration of a trial was 100 s, subjecting the subject to 25 rotations to the right and 25 rotations to the left. The frequency and amplitude characteristics were determined in order for the platform movements to destabilize posture (i.e., lateral tilt) and demand that subjects relied on anticipatory postural adjustments to counteract for the destabilization. Higher frequencies and amplitudes would have not been safely sustainable by the young infants. Further, higher frequency and amplitude oscillations would have produced a too large variability in the response behaviour of the subjects and would have induced neuromuscular fatigue with the potential of confounding the postural adjustments. Conversely, lower frequencies and amplitudes would have not implied enough postural destabilization, leading to small postural adjustments that are difficult to measure.

### Procedures

Once arrived in the laboratory, the subject was provided a few minutes to become acclimated to the testing environment and experimenters. The laboratory simulated a common living room intended to provide calm and soothing environment for the subject. Following this period, upper body clothing and pants were removed in order for the experimenters to place electrodes and reflective markers. Bipolar surface electrodes (dimensions 15 mm×15 mm, inter-electrode distance 20 mm) were placed over the surface of lower back and neck muscles, namely the erector spinae (ES) muscle (30 mm lateral to the spinal process of the second lumbar vertebra) and the trapezius (T) muscle (over its cervical part). Reflective markers were placed on several anatomical landmarks of the upper body and head including the head vertex, the mastoid processes, the spinal processes of the seventh cervical vertebra, the sixth and the twelfth thoracic vertebrae, the second lumbar vertebra, the sacrum, and the posterior superior iliac spines (Fig. 1A). Body segments were subsequently defined from the markers (Fig. 1B). Two additional markers were also placed on the platform.

The subject then sat on the platform in a self-selected position. The experimenters made sure that the subject was positioned along the axis of rotation of the platform with the legs stretched forward and the arms rested on the legs (Fig. 1A). The subject then underwent 100 s rotations. The platform was located 1.5 m away from a white wall, preventing the visual attention of the subjects from being oriented toward a particular location in the visual field. No instructions were provided to adult subjects as to how they should respond to the platform oscillations in order to place them in similar experimental conditions than infants and measure spontaneous postural responses and kinematic adaptations.

Electromyograms (EMGs) were recorded with a TELEMG multi-channel system (BTS, Milan, Italy). The EMG signals were pre-amplified (5,000×), analog bandpass-filtered between 20 and 450 Hz, acquired at a sampling frequency of 1,000 Hz and stored on a PC for off-line analysis. The three-dimensional positions of the markers were acquired at 100 Hz with an ELITE automatic motion analyzer (BTS, Milan, Italy).

### Analysis

#### Emg

**Figure 2:**
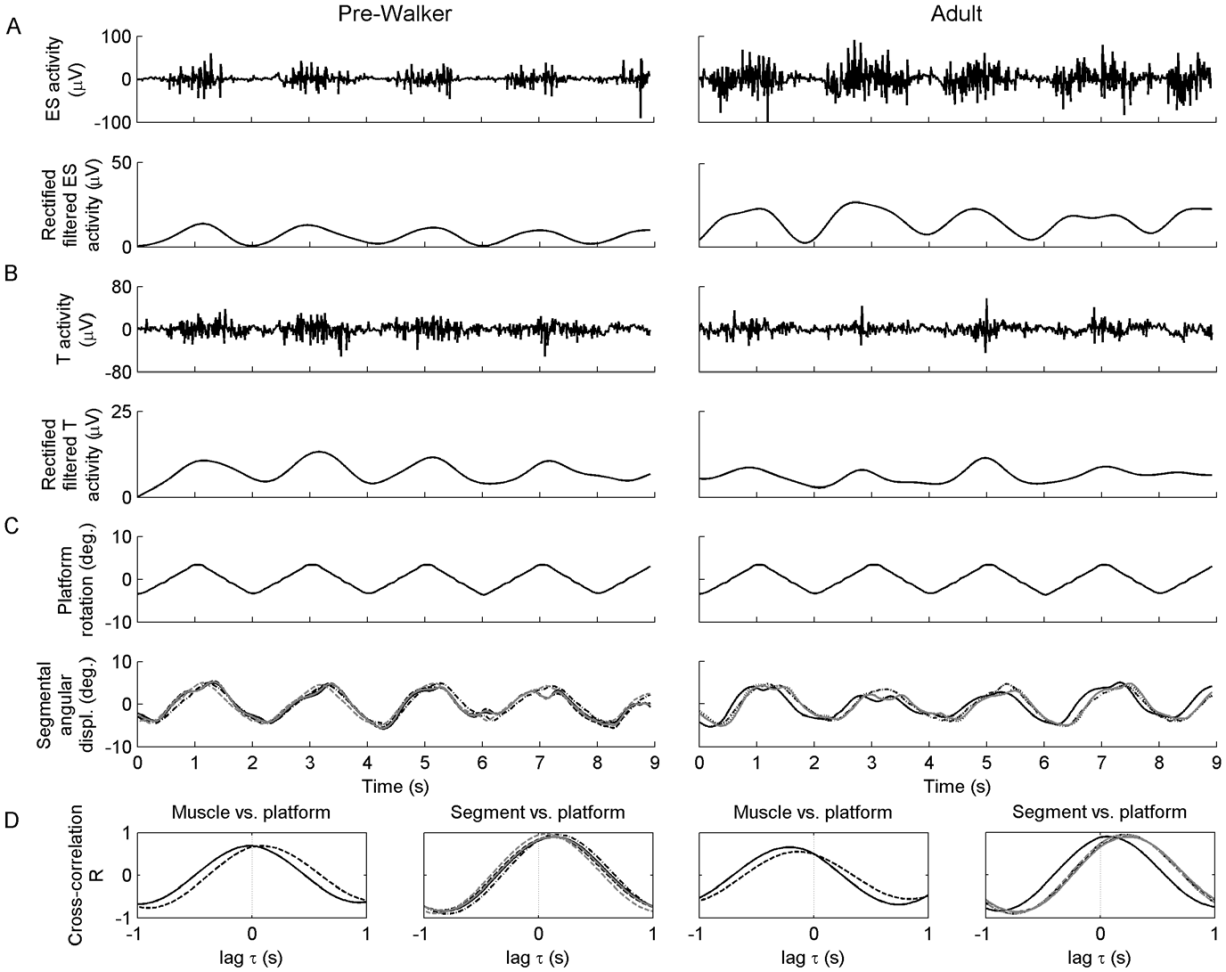
Illustration of data analysis in a pre-walker and an adult. (A) Typical raw EMGs from erector spinae (ES) and trapezius (T) muscles. The raw signals were then rectified and filtered to obtain envelopes of the EMG patterns. (B) Typical patterns of platform rotation and angular displacements of body segments. (C) Cross-correlation functions between muscle activity or segmental angular displacement and platform rotation. A negative time-lag at the maximum cross-correlation indicates that either the peak of muscle activity or the turning point of segment oscillation precedes the turning point of the platform, and vice versa for a positive-time lag.

The signals were bandpass filtered from 25 to 250 Hz using a fourth-order bidirectional (i.e., zero-lag) Butterworth filter, bandstop filtered from 57–63 Hz to remove any residual 60 Hz noise [20], [21], and full-wave rectified. These pre-processed EMG signals were then filtered with a low-pass bidirectional Butterworth filter of order four with a cut-off frequency of 1 Hz, producing an integrated EMG (Fig. 2A). Note that this filtering outlined the envelope of the EMG pattern while it cancelled small non-recurrent changes in it, which were detrimental to subsequent analysis. Finally, the integrated EMG signals were re-sampled from 1,000 to 100 Hz so that they had the same length as kinematic signals.

#### Kinematics

The raw marker data were filtered with a fourth-order zero-lag Butterworth filter with a cutoff frequency of 10 Hz. The angular displacements of the segments head, upper- and lower-torso, lumbar and pelvis together with the angular displacement of the platform were afterwards obtained in the frontal plane from the filtered markers’ positions (Fig. 2B).

#### Cross-Correlation

The coupling between the periodic activation of the ES and T muscles and the platform rotation was determined using sample cross-correlation estimation [22]:
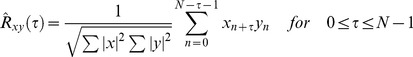
(1)


and
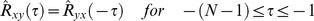
where 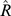 and *τ* denote the estimate of the correlation coefficient and the time lag between data sets *x* and *y* of length *N*, respectively; *x* representing the integrated EMG and *y* the angular displacement of the platform. Note that cross-correlation was normalized so that in case *x* and *y* would have been similar, the correlation coefficient at lag 0 would have been 1. Technically, Eq. 1 was solved using a fast Fourier transform (FFT) procedure, which was more efficient than solving it directly [22], [23]. The 100-s-long *x* and *y* data sets were first segmented into several overlapping segments that counted 2^15^ points (i.e., segments 32.768 s long). Then, 15-bit FFT algorithm was applied to these segments to generate segments’ periodograms that were averaged to obtain an ‘ensemble average’ power spectral density of improved statistical stability. Finally, cross-correlation was obtained by applying the inverse FFT to the cross-power spectral density function of *x* and *y*, which was obtained from the product of the FFT transforms of *x* and *y*. The maximum value of 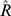 was used as the variable of interest to evaluate the strength of the association between the integrated EMGs and the platform movement, the higher the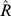-value the stronger the association (Fig. 2C). Another variable of interest was the time lag *τ* at which the maximum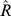-value occurred (Fig. 2C). A lag of 0 indicates a perfect in-phase relationship between the integrated EMG and the platform; a positive lag indicates that the integrated EMG is delayed with respect to the platform and inversely for a negative lag. Both a negative lag and a lag of 0 were interpreted as expressing postural anticipation [24], [25]. Specifically, each previous variable was averaged over the right- and left-side muscles for each subject. Moreover, cross-correlation estimations were also conducted between angular displacement of body segments and platform movement to determine which segments were most strongly related to the support (Fig. 2C). In all cases, the accuracy of the time delay calculation was 10 ms, due to the sampling frequency of 100 Hz.

#### Marker displacement

The displacements of the markers in the medial-lateral direction at the time of maximal rotation of the platform to the right and to the left were averaged over the trial to get information about the strategy of balance control. These displacements were calculated with respect to initial position before the platform start rotating, the lower the averaged displacement the less destabilized the upper body (i.e., a good damping of the perturbation). Given that averaged medial-lateral displacement strongly depended upon the subject’s upper-body height, group data were normalized by removing trends due to height [26], [27]. Specifically, marker data sets for each group of subjects were fitted in a least-squares sense using first-order polynomials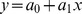. Detrended displacements, 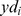, were then obtained from original data 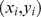 as:
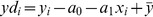
(2)where the index *i* represents any given subject and 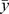 is the mean value of the original data set. Therefore, the transformed medial-lateral displacements data were no longer correlated with upper-body height and were scaled to a similar range as the original data.

#### Angular dispersion

Further information on the damping of the perturbation about the body segments was obtained from the averaged angular dispersion (i.e., standard deviation of angular displacement) of the body segments at the time of maximal rotation of the platform [28], [29]. The lower the averaged angular dispersion of a given segment, the more attenuated the perturbation about it.

#### Statistics

Kruskal-Wallis one-way analysis of variances (ANOVAs) and Friedman repeated measures ANOVAs were used to examine differences in dependant variables between groups and repeated conditions, respectively. When ANOVAs yielded significant results, *post hoc* assessment was performed by Dunn’s multiple comparison tests and multiple comparison correction by a step-down Bonferroni-Holm procedure. One-sample Wilcoxon signed rank tests were also used to evaluate whether time lags *τ* differed from 0. Statistical analysis was performed using SigmaStat statistical software package v.4. The threshold of statistical significance was set at p<0.05. In case of multiple comparisons, this threshold applied for the whole family of comparisons and the per-comparison significance was evaluated from corrected p-values.

## Results

### Coupling between Integrated EMGs and Platform

**Figure 3:**
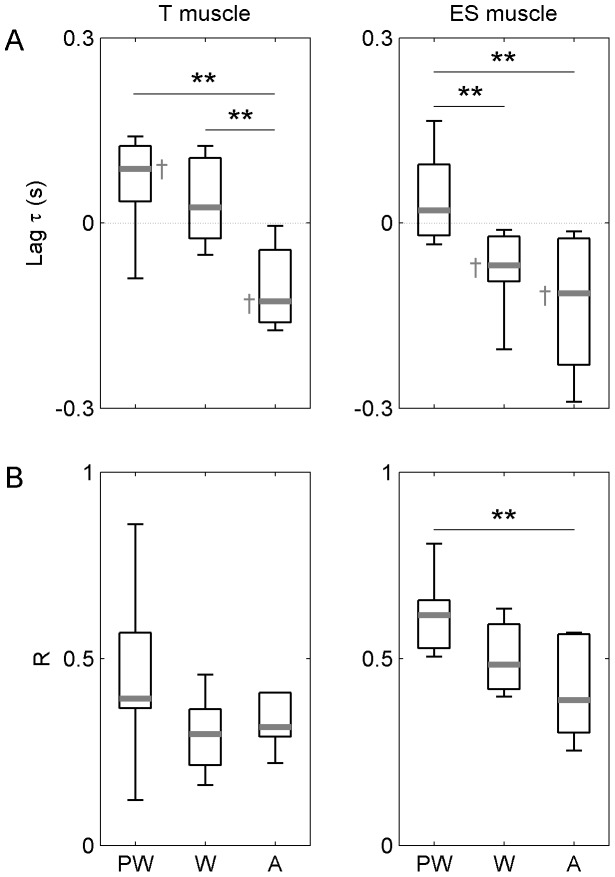
Cross-correlation between muscle activation and platform movement in infants at the pre-walking stage (PW), infants who attained independent walking (W), and young adults. (A) Lag τ between muscle activation and platform movement. (B) Peak correlation R for muscle activation and platform movement. Data are presented as median (grey mark) and interquartile range (black box). Whiskers extend to the data’s minimum and maximum. Group differences from pairwise multiple comparisons (Dunn’s tests associated with a Bonferroni-Holm correction) are reported using horizontal bars. **: p-value <0.01.

As can be seen on figure 3A, the lags *τ* between EMGs and platform movement exhibited differences between groups, with a significant group effect revealed by Kruskall Wallis ANOVAs for both the ES muscle, H(2, N = 26) = 12.17, p = 0.002, and the T muscle, H(2, N = 26) = 11.67, p = 0.003. On the one hand, one-sample Wilcoxon signed rank tests indicated that the time lag for the ES muscle in infants at the pre-walking stage (2±10 ms) was not statistically different from zero, Z = 0.38, p>0.05, while the time lags in walking infants (−68±67 ms) and young adults (−114±180 ms) were significantly lower than zero, Z = −0.72 and Z = −0.78, respectively, p<0.05. This difference between groups was confirmed using pairwise multiple comparisons, with the time lag in infants at the pre-walking stage significantly greater than the time lags in walking infants and young adults, Q = 2.81, p<0.01, and Q = 2.97, p<0.01, respectively. Therefore, the activation bursts for the ES muscle and the turning points of the platform occurred at the same time in infants who had not attained independent walking yet whereas the bursts started before the time of occurrence of the turning points in the other two groups. This result indicated that walking infants and young adults had ability to activate ES muscle in anticipation of platform movements while infants who did not acquire independent walking only demonstrated premises in anticipation. In addition, a group effect was found on the peak correlation
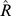
of the cross-correlation function, H(2, N = 26) = 9.54, p = 0.008, with a higher correlation in infants at the pre-walking stage (0.61±0.11) as compared to adults (0.38±0.22), Q = 2.97, p<0.01 (Fig. 3B). Thus, the strength of the covariation between the EMG and the platform movement decreased in adults.

On the other hand, a different pattern of findings was observed for the T muscle (Fig. 3A). The one-sample Wilcoxon signed rank tests indicated that the time lag in infants at the pre-walking stage (87±84 ms) was statistically greater than zero, Z = 0.72, p<0.05, while the time lag in walking infants (2±119 ms) did not differ from zero, Z = 0.38, p>0.05, and the adults’ time lag (−127±91 ms) was significantly lower than zero, Z = −0.64, p<0.05. Further examination of the data using pairwise multiple comparisons indicated that the two groups of infants exhibited significantly higher lags than adults, Q = 3.46, p<0.01 (pre-walkers vs. adults) and Q = 2.45, p<0.01 (walkers vs. adults). Thus, there was a clear anticipation of platform movements by the T muscle activity in young adults and a beginning ability in infants who already walked independently. Finally, no group effect was observed on peak correlation (Fig. 3B).

### Coupling between Segments and Platform

**Figure 4:**
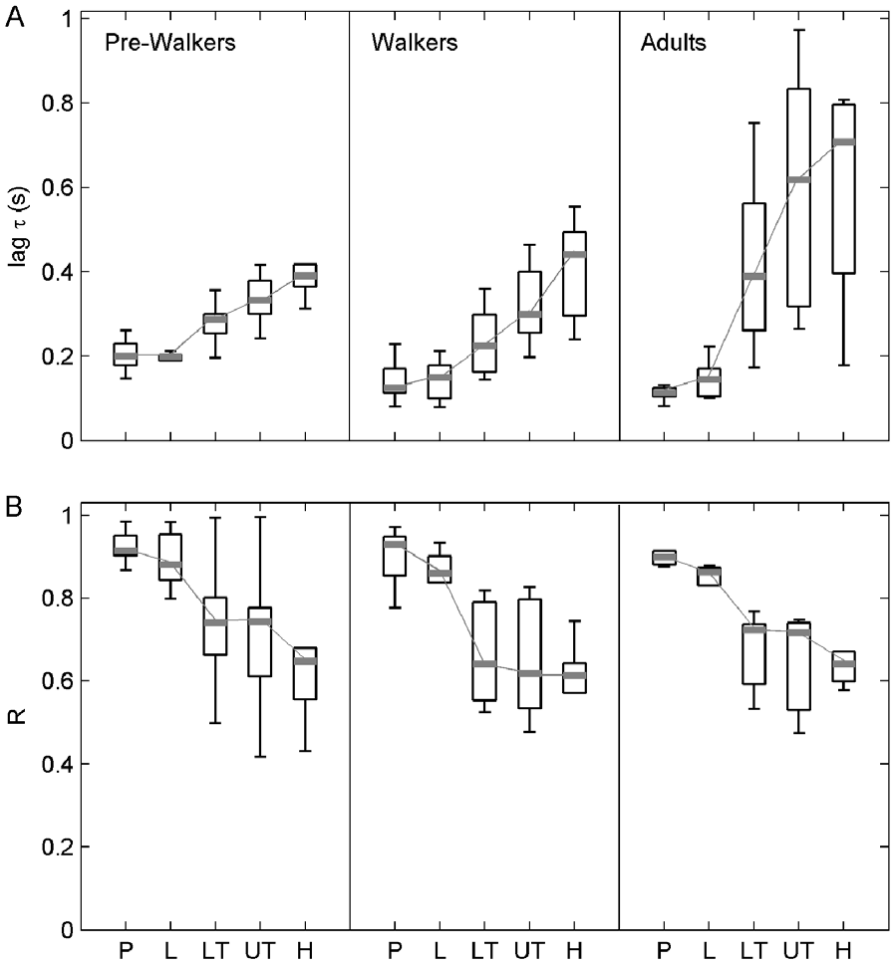
Cross-correlation between segmental movement and platform movement in walking and non-walking infants and young adults. (A) Lag τ between segmental movement and platform movement. (B) Peak correlation R for segmental movement and platform movement. Data are presented as median (grey mark) and interquartile range (black box). Whiskers extend to the data’s minimum and maximum. P: pelvis, L: lumbar segment, LT: lower-torso, UT: upper-torso, H: head.

Lags *τ* in all three groups were significantly higher than 0, meaning that segmental displacements followed platform rotation. Friedman repeated measures ANOVAs that were conducted on lag *τ* revealed significant differences in temporal coupling between the body segments and the platform in pre-walking infants, χ^2^(4, *N* = 10) = 35.84, p<0.001, walking infants, χ^2^(4, *N* = 10) = 38.08, p<0.001, and adults, χ^2^(4, *N* = 10) = 21.01, p<0.001. Multiple paired comparisons showed that the turning points of the head and the upper-torso oscillations were more delayed than those of the lumbar segment and the pelvis with respect to the turning points of the platform in all three groups of subjects, 2.83< Q <5.11, 0.001<p<0.01 (Fig. 4A). In addition, Friedman repeated measures ANOVAs that were conducted on the peak correlation 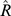
revealed significant differences between body segments in pre-walking infants, χ^2^(4, *N* = 10) = 27.21, p<0.001, walking infants, χ^2^(4, *N* = 10) = 33.68, p<0.001, and adults, χ^2^(4, *N* = 10) = 15.61, p<0.004. Specifically, multiple testing revealed that the coupling strength with the platform was larger for the lower body segments (i.e., pelvis and lumbar segment) than for the higher body segments (i.e., head and upper torso) in all groups, 3.11< Q <4.95, 0.001<p<0.01 (Fig. 4B). Taken together, the above results thus indicated that the higher body segments were more independent from platform movement than the lower body segments.

Moreover, Kruskall Wallis ANOVAs revealed a significant group effect for lag *τ* between platform movement and segmental movement for the lower-torso, H(2, N = 26) = 6.58, p = 0.037, the upper torso, H(2, N = 26) = 9.41, p = 0.009, and the head, H(2, N = 26) = 5.75, p = 0.043. Lags were higher in adults (lower-torso: 389±281 ms; upper-torso: 617±472 ms; head: 706±340 ms) than in infants at the pre-walking stage (lower-torso: 285±42 ms; upper-torso: 331±68 ms; head: 389±52 ms) as revealed by multiple comparisons, 2.41< Q <3.04, 0.01<p<0.05 (Fig. 4A). Therefore, the higher body segments were more independent from platform movement in adults than in non-walking infants. Finally, there was no group effect on peak correlation for all segments (Fig. 4B).

### Normalized Marker Lateral Displacement

**Figure 5:**
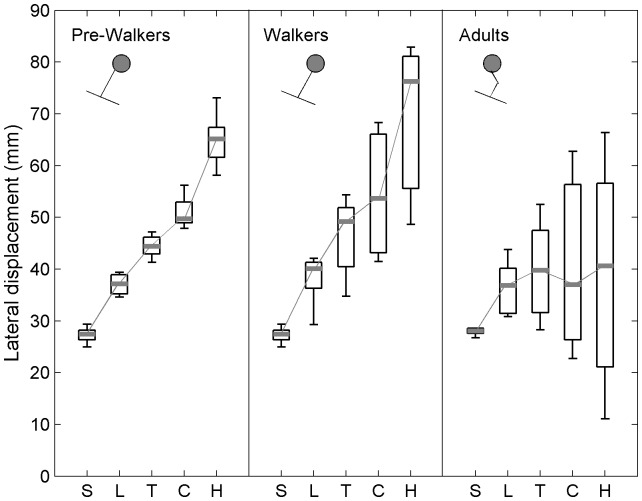
Normalized lateral displacements of the anatomical markers in infants at the pre-walking stage (pre-walkers), walking infants (walkers), and adults. Data are presented as median (grey mark) and interquartile range (black box). Whiskers extend to the data’s minimum and maximum. S: sacrum marker, L: lumbar marker, T: thoracic marker, C: cervical marker, H: head marker. Results are illustrated using stick figures (top left corner).

Significant differences in displacement across markers were found for both infants at the pre-walking stage, χ^2^(4, *N* = 10) = 40, p<0.001, and walking infants, χ^2^(4, *N* = 10) = 40, p<0.001. Multiple paired comparisons revealed in both groups an increase of lateral displacement from lower markers to higher markers. Specifically, the lateral displacement of the head marker was found larger than that of the thoracic marker, which was also found larger than that of the pelvis marker, 2.82< Q <5.65, 0.001<p<0.05. Inversely, the markers of the young adults were all found at the same distance from initial position, with an insignificant Friedman test result, χ^2^(4, *N* = 6) = 5.86, p = 0.21 (Fig. 5). Therefore, the young adults remained rather upright, while the infants tilted in congruence with the platform with their upper body behaving like a rigid inverted pendulum.

Kruskall-Wallis ANOVAs provided further information on the above difference between adults and infants regarding the ability to remain upright on the moving platform. While no difference between the three groups was observed for the lateral displacement of the sacrum, lumbar, thoracic and cervical markers, a group effect was found for the head marker, H(2, N = 26) = 7.88, p = 0.019. Multiple testing indicated that the lateral displacement of the head marker was significantly lower in adults as compared to infants who did, Q = 2.61, p<0.01, and did not attained, Q = 2.53, p<0.01, independent walking (Fig. 5). Therefore, this result suggests that the adults adopted a head stabilization in space strategy while the infants did not.

### Segmental Angular Dispersion

**Figure 6:**
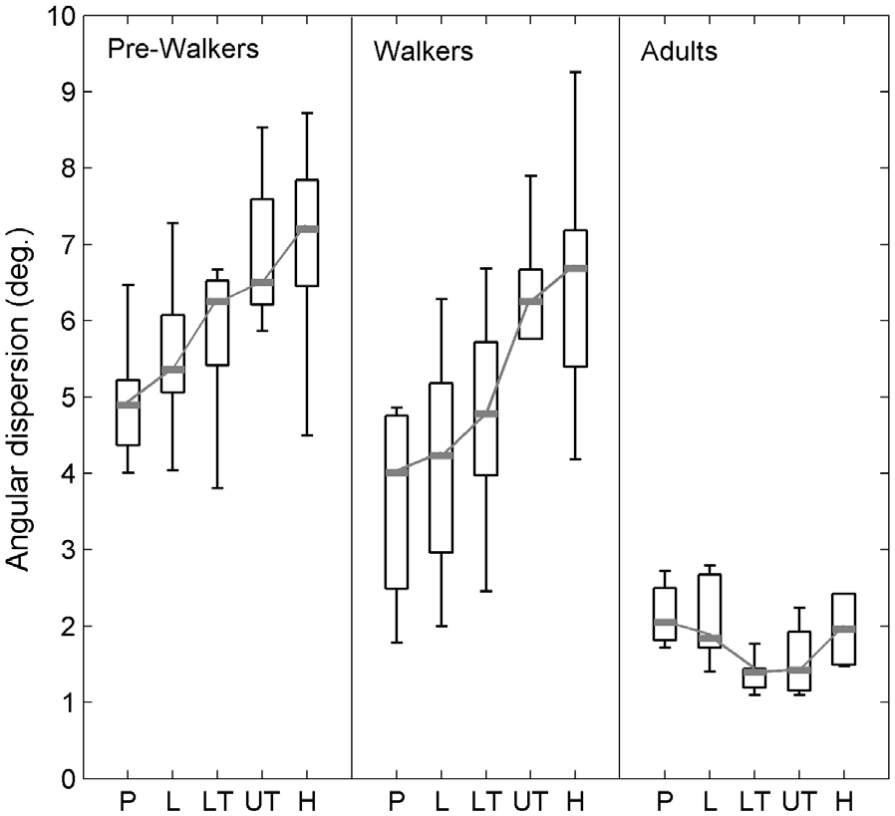
Angular dispersions of the body segments in infants at the pre-walking stage (pre-walkers), walking infants (walkers), and adults. Data are presented as median (grey mark) and interquartile range (black box). Whiskers extend to the data’s minimum and maximum. P: pelvis, L: lumbar segment, LT: lower torso, UT: upper torso, H: head.

Within-group analysis of segmental dispersion showed differences between segments in infants at the pre-walking stage, χ^2^(4, *N* = 10) = 20.56, p<0.001, and walking infants, χ^2^(4, *N* = 10) = 33.84, p<0.001. On the other hand, no difference was revealed in young adults, χ^2^(4, *N* = 6) = 8.91, p = .07. In infants at the pre-walking stage, paired comparisons revealed that the angular dispersions of the head and upper-torso segments were significantly larger than that of the pelvis, Q = 3.67, p<0.01, and Q = 3.54, p<0.01, respectively. In walking infants, the angular dispersions of the head and upper-torso segments were also significantly larger than that of the pelvis, Q = 4.38, p<0.01, and Q = 4.24, p<0.01, respectively, and that of the lumbar segment, Q = 3.96, p<0.01, and Q = 3.81, p<0.01, respectively (Fig. 6). Thus, the perturbations from the platform were attenuated about anatomical segments in young adults while they propagated as going up to higher segments in infants.

Between-group analysis of the segmental dispersion indicated a significant group effect for all the segments: pelvis, H(2, N = 26) = 14.97, p<0.001, lumbar segment, H(2, N = 26) = 14.49, p<0.001, lower torso, H(2, N = 26) = 15.47, p<0.001, upper torso, H(2, N = 26) = 14.44, p<0.001, and head, H(2, N = 26) = 14.32, p<0.001. Multiple testing indicated that the angular dispersions in infants at the pre-walking stage were significantly larger than those in adults for all segments, 3.92≤ Q ≤3.72, p<0.01. The angular dispersions in walking infants were also significantly larger than those in adults for the upper segments (i.e., lower torso, upper torso, and head), 2.65≤ Q ≤2.86, p<0.01 (Fig. 6). Accordingly, walking infants demonstrated an adult-like ability to attenuate platform perturbations only about lower segments.

## Discussion

Controlling actions prospectively is a major achievement for infants. Although anticipatory postural adjustments start developing as soon as the first year of life, there has been suggestive evidence that they become more consistent once independent walking is acquired [10]–[12]. The present study was therefore intended to provide further evidence that independent walking experience plays a role in the development of anticipatory postural control. Specifically, it was expected that walking infants would show greater sophistication compared to non-walking infants in their adult-like ability to anticipate for postural disturbances induced by the moving platform.

### Anticipatory Postural Adjustments to Platform Rotation: What is Changed with Independent Walking and why does it Change?

There were two main results with respect to anticipatory control, as evaluated from muscular adjustments intended to stabilize posture against platform disturbances. On the one hand, infants who did not walk independently demonstrated premises for anticipatory adjustments about the low back (i.e., ES muscle) only. On the other hand, infants who were already walking alone demonstrated ability for anticipatory adjustments about the low back and the neck (i.e., T muscle), including an adult-like ability at the former level and premises for anticipation at the latter level. Therefore, in line with what we had expected, independent walking is not a prerequisite for anticipatory postural control. However, once independent walking has been acquired, a robust, adult-like, ability to anticipate for postural perturbations emerges. These results thus complement previous works that have proposed independent walking as an essential transition point for the development of anticipatory postural control [10]–[12].

It is noteworthy that anticipatory adjustments to platform rotation developed in a bottom-up fashion, occurring first at the low back and then developing from the low back to the neck from independent walking onwards. This finding questions the maturational viewpoint that supports a cephalo-caudal gradient in the development of postural responses during the first year of life. Indeed, experiments that have examined postural adjustments when perturbing the support surface or performing reaching movement rather reported that infants by the end of the first year of life prefer to activate neck muscles first [7], [10], [30], [31], which afterwards disappear for a bottom-up recruitment as observed in sitting children [32] and sitting and standing adults [33]–[35]. This outcome difference may come from the fact that instead comparing postural adjustments in infants of different ages, we considered this issue by grouping infants based on the skills they were mastering (i.e., standing and walking). Considering change over time on such basis, Assaiante and Amblard [36] proposed that postural control (i) becomes organized in a bottom-up way from the time upright posture is acquired, and (ii) aims at stabilizing the pelvis to minimize the displacements of the center of gravity from walking onset. As regards the latter point, we did observe a better stabilization (i.e., more attenuation of the perturbation as evaluated from angular dispersion) of the pelvis in walking infants and adults as compared to non-walking infants, supporting the idea that from the moment walking is acquired, postural control (here anticipatory) strongly stabilizes the low back. Thus, it turns out that the present results are in line with an experience-driven explanation for developmental progress [37], meaning that experience maintaining balance in a variety of postures (standing and walking) is a facilitator to the development of anticipatory postural adjustments. Interestingly, the moderate ability to compensate in advance for the perturbations about the pelvis before independent walking suggests that anticipatory postural control may be also a facilitator to it. In particular, anticipatory muscle activity is required during walking to stabilize pelvis and organize balance control [15], [38]. Therefore, walking may be a facilitator to anticipatory postural control and vice versa, reinforcing each other as proposed by Barela et al. [12].

However, the significance of walking experience on the development of anticipatory postural control should be considered with caution since most of the non-walking infants were younger than the walking infants, so that age was confounded with walking experience in the study. Thus, disentangling the role of age from that of walking experience is critical to definitely establish a link between walking and developmental changes in anticipatory postural control. Previous studies on the role of crawling experience in changes in cognitive, social and emotional development are instructive in this regard (see [39] for a review). These studies have established the experimental designs needed to isolate the role of locomotor experience as a facilitator of development, including for example age-held constant designs (i.e., holding infant age constant while examining variations in walking experience) and lag-sequential designs (i.e., permitting the assessment of the role of age, locomotor experience, and their interaction). Therefore, future studies should use designs of this nature to infer the role of independent walking in the development of anticipatory postural adjustments.

### ‘Balancing’ Behaviour to Platform Rotation: What Sort of Pendulum?

An important result was that infants, even the walking ones who demonstrated clear anticipatory adjustments about the low back, did not remain upright, tilting with the platform (i.e., a tight coupling to the platform). This behaviour was reflected in lateral displacements and time lags between the platform and the body segments that progressively increased as going up to the most distal body segment (i.e., the head). Inversely, adults remained more upright and more independent from platform rotations with respect to their distal body segments. Indeed, lateral displacements remained similar for all markers and the time lags between the platform and the lower-torso, the upper-torso, and the head were rather large, reflecting an articulated operation of the head-trunk unit [36]. These findings tend to demonstrate that infants behaved as rigid inverted pendulum with a mass and a spring, its base being attached to the rotating platform, while upper body movements in adults rather resembles that of a non-rigid, non-inverted pendulum rotating about the mass. In other words, while infant were following the platform and were thus counteracting body inertia too late when the platform already went through its turning points, adults learned to counteract body inertia between the two end-points of rotation of the platform to stabilize head in space. This behaviour in adults agrees with previous studies where subjects who were standing on a continuously anterior-posterior translating platform were damping perturbations by stabilizing trunk and head to create a pivot for the passive displacements of the lower limbs, entrained by the platform displacement [17]–[19].

However, what are the origins of such difference between infants and adults? Corna et al. [17] reported a transition from a non-rigid, non-inverted pendulum to a rigid inverted pendulum strategy when visual inputs were removed in adults standing on a moving platform, concluding that the central nervous system may use visual information in a feedforward manner to reduce head oscillation in space. Isableu et al. [40] also evidenced that visual field dependant subjects adopted an “en bloc” functioning (i.e., behaved like rigid inverted pendulum) in the absence of adequate visual cues when remaining upright, suggesting that they blocked their joints due to difficulty in using corresponding proprioceptive cues. Although we did observe inverted pendulum behaviour in infants, neither an inability to perceive optical flow nor an inability to use proprioceptive cues or an inability to generate anticipatory motor commands from both sources of information likely explain difference with adult behaviour. First, there is extensive evidence in the literature that infants in the second half of the first year of life are able to perceive optical flow and scale their postural responses to the visual information [41], [42]. Besides, infants should have been also able to interiorise the platform rhythm on the basis of the other sensory inputs, namely the vestibular and the somatosensory inputs. Indeed, it has been established that the vestibular system, which has an important role in balance control [43], is already very sensitive during the first 6 months of life [44]. With respect to somatosensation, Hadders-Algra et al. [30] demonstrated that compensatory muscle activations, likely triggered by somatosensory signals from the pelvis, already occurred in 5-month-old sitting infants to bring upper body back to vertical once destabilized by a moveable platform. Second, we observed in both pre-walkers and walkers an ability for postural anticipation about muscles, which reflects their ability to integrate multiple inputs (here, visual, somatosensory and vestibular inputs) and use them feedforwardly. Therefore, a remaining explanation for the infants to behave like a rigid inverted pendulum, not being able to stabilize upper-body and head in space, is that priori knowledge of the platform effect on sitting posture was not accurate, so that motor commands sent to the musculature to maintain the upright posture were not appropriate to platform perturbations (i.e., an inappropriate scaling between platform effect and postural command). This latter outcome likely results from immaturity of the central nervous system to update internal model that integrates both body characteristics and external perturbation, as discussed below.

### Building Internal Model Combining Postural Experience and External Perturbation

When movement prediction depends on both the individual body and an external object, as here the platform, not only does the central nervous system build an internal model of the object but it also integrates the dynamics of that object into an internal model of the sensorimotor system as a whole [45], [46]. In this view, the anticipatory adjustments observed about the low back muscle in infants having not walked yet indicate that this process already took place before independent walking, although it remained importantly immature. In walking infants, this internal model of the sensorimotor system and platform appeared to be more accurate. Anticipation occurred about both the low back and neck muscles and there was also kinematic adjustment at the low back level that attenuated, in an adult-like way, the platform perturbations. Thus, it is likely that the variety of everyday walking experience enriched the internal model of the sensorimotor system and facilitated integrating the dynamics of the platform within it, although this latter element remained difficult as suggested by the infants’ inability to remain upright.

Taken together, results from the present study thus support the idea that postural behaviours underlying motor skills share a similar internal model for postural control. This is in contrast to studies by Adolph [47], [48] who rather concluded on the absence of sensorimotor learning transfer between the postural milestones (e.g., sitting, standing, crawling, and walking). On the other hand, our suggestion accords with a recent work by Chen et al. [49], which emphasized that any new behavior is built upon some of the basic control elements used in managing the previously learned behavior, enriching the existing internal model.

In conclusion, our study indicated that although independent walking is not a prerequisite for postural anticipation, adult-like anticipation ability seems to emerge once this skill is acquired. However, other research designs controlling for age effects are needed to firmly establish independent walking *per se* as a facilitator to the development of anticipatory postural control. With regard to the behaviour adopted by the subjects to maintain balance during the continuous rotation of the platform, infants contrary to adults had difficulties to stabilize upper body and head in space, likely due to an inaccurate estimation of the platform effects on posture. These findings support the idea that postural behaviour relied on an internal model that included the sensorimotor system and the platform, with difficulty in infants for integrating the dynamics of the platform within the model.

## References

[1] von Hofsten C (2004) An action perspective on motor development. Trends Cogn Sci 8: 266–272. 15165552 10.1016/j.tics.2004.04.002

[2] von Hofsten C (1993) Prospective control: A basic aspect of action development. Hum Dev 36: 253–270.

[3] Assaiante C, Mallau S, Viel S, Jover M, Schmitz C (2005) Development of postural control in healthy children: a functional approach. Neural Plast 12: 109–118. 16097479 10.1155/NP.2005.109 PMC2565455

[4] Aruin AS, Latash ML (1995) Directional specificity of postural muscles in feed-forward postural reactions during fast voluntary arm movements. Exp Brain Res 103: 323–332. 7789439 10.1007/BF00231718

[5] Massion J (1992) Movement, posture and equilibrium: interaction and co-ordination. Progr Neurobiol 38: 35–56. 10.1016/0301-0082(92)90034-c 1736324

[6] Nardone A, Schieppati M (1988) Postural adjustments associated with voluntary contraction of leg muscles in standing man. Exp Brain Res 69: 469–480. 3371431 10.1007/BF00247301

[7] de Graaf-Peters VB, Bakker H, van Eykern LA, Otten B, Hadders-Algra M (2007) Postural adjustments and reaching in 4- and 6-month-old infants: an EMG and kinematical study. Exp Brain Res 181: 647–656. 17505820 10.1007/s00221-007-0964-6

[8] van Balen LC, Dijkstra LJ, Hadders-Algra M (2012) Development of postural adjustments during reaching in typically developing infants from 4 to 18 months. Exp Brain Res 20: 109–119. 10.1007/s00221-012-3121-9 PMC3380253 22623096

[9] van der Fits IBM, Hadders-Algra M (1998) The development of postural response patterns during reaching in healthy infants. Neurosci Biobehav Rev 22: 521–526. 9595564 10.1016/s0149-7634(97)00039-0

[10] van der Fits IBM, Otten E, Klip AWJ, van Eykern LA, Hadders-Algra M (1999) The development of postural adjustments during reaching in 6- to 18-month-old infants. Exp Brain Res 126: 517–528. 10422715 10.1007/s002210050760

[11] Witherington DC, von Hofsten C, Rosander K, Robinette A, Woollacott MH, et al (2002) The development of anticipatory postural adjustments in infancy. Infancy 3: 495–517.

[12] Barela JA, Jeka JJ, Clark JE (1999) The use of somatosensory information during the acquisition of independent stance. Infant Behav Dev 22: 87–102.

[13] Assaiante C, Thomachot B, Aurenty R (1993) Hip stabilization and lateral balance control in toddlers during the first four months of autonomous walking. Neuroreport 4: 875–878. 8369477 10.1097/00001756-199307000-00009

[14] Assaiante C, Thomachot B, Aurenty R, Amblard B (1998) Organization of lateral balance control in toddlers during the first year of independent walking. J Motor Behav 30: 114–129. 10.1080/00222899809601329 20037027

[15] Assaiante C, Woollacott MH, Amblard B (2000) Development of postural adjustment during gait initiation: Kinematic and EMG analysis. J Motor Behav 32: 211–226. 10.1080/00222890009601373 10975270

[16] Buchanan JJ, Horak FB (1999) Emergence of postural patterns as a function of vision and translation frequency. J Neurophysiol 81: 2325–2339. 10322069 10.1152/jn.1999.81.5.2325

[17] Corna S, Tarantola J, Nardone A, Giordano A, Schieppati M (1999) Standing on a continuously moving platform: is body inertia counteracted or exploited? Exp Brain Res 124: 331–341. 9989439 10.1007/s002210050630

[18] De Nunzio AM, Schieppati M (2007) Time to reconfigure balancing behaviour in man: changing visual condition while riding a continuously moving platform. Exp Brain Res 178: 18–36. 17013618 10.1007/s00221-006-0708-z

[19] Schieppati M, Giordano A, Nardone A (2002) Variability in a dynamic postural task attests ample flexibility in balance control mechanisms. Exp Brain Res 144: 200–210. 12012158 10.1007/s00221-002-1028-6

[20] DeLuca CJ (1997) The use of surface electromyography in biomechanics. J Appl Biomech 13: 135–163.

[21] Dingwell JB, Joubert JE, Diefenthaeler F, Trinity JD (2008) Changes in muscle activity and kinematics of highly trained cyclists during fatigue. IEEE Trans Biomed Eng 55: 2666–2674. 18990638 10.1109/TBME.2008.2001130 PMC2905840

[22] Orfanidis SJ (1996) Optimum Signal Processing. An Introduction. 2^nd^ Edition, Englewood Cliffs, NJ: Prentice-Hall.

[23] Bloomfield P (2000) Fourier Analysis of Time Series. Toronto: Wiley.

[24] Santos MJ, Kanekar N, Aruin AS (2010) The role of anticipatory postural adjustments in compensatory control of posture: 1. Electromyographic analysis. J Electromyogr Kinesiol 20: 388–397. 19660966 10.1016/j.jelekin.2009.06.006

[25] Shiratori T, Latash ML (2001) Anticipatory postural adjustments during load catching by standing subjects. Clin Neurophysiol 112: 1250–1265. 11516737 10.1016/s1388-2457(01)00553-3

[26] O’Malley MJ (1996) Normalization of temporal-distance parameters in pediatric gait. J Biomech 29: 619–625. 8707788 10.1016/0021-9290(95)00088-7

[27] Chiari L, Rocchi L, Cappello A (2002) Stabilometric parameters are affected by anthropometry and foot placement. Clin Biomech 17: 666–677. 10.1016/s0268-0033(02)00107-9 12446163

[28] Assaiante C, Amblard B (1993) Ontogenesis of head stabilization in space during locomotion in children: Influence of visual cues. Exp Brain Res 93: 499–515. 8519339 10.1007/BF00229365

[29] Mallau S, Vaugoyeau M, Assaiante C (2010) Postural strategies and sensory integration: no turning point between childhood and adolescence. PLoS One 5: pii: e13078. 10.1371/journal.pone.0013078 PMC2947520 20927328

[30] Hadders-Algra M, Brogren E, Forssberg H (1996a) Ontogeny of postural adjustments during sitting in infancy: variation, selection and modulation. J Physiol 493: 273–288. 8735712 10.1113/jphysiol.1996.sp021382 PMC1158968

[31] Hadders-Algra M, Brogren E, Forssberg H (1996b) Training affects the development of postural adjustments in sitting infants. J Physiol 493: 289–298. 8735713 10.1113/jphysiol.1996.sp021383 PMC1158969

[32] Brogren E, Hadders-Algra M, Forssberg H (1996) Postural control in children with spastic diplegia: muscle activity during perturbations in sitting. Dev Med Child Neurol 38: 379–388. 8698146 10.1111/j.1469-8749.1996.tb15095.x

[33] Horak FB, Nashner LM (1986) Central programming of postural movements; adaptation to altered support-surface configurations. J Neurophysiol 55: 1369–1381. 3734861 10.1152/jn.1986.55.6.1369

[34] Forssberg H, Hirschfeld H (1994) Postural adjustments in sitting humans following external perturbations: muscle activity and kinematics. Exp Brain Res 97: 515–527, 1994. 10.1007/BF00241545 8187862

[35] van der Fits IBM, Klip AWJ, Van Eykern LA, Hadders-Algra M (1998) Postural adjustments accompanying fast pointing movements in standing, sitting, and lying adults. Exp Brain Res 120: 202–216. 9629962 10.1007/s002210050394

[36] Assaiante C, Amblard B (1995) An ontogenetic model for the sensorimotor organization of balance control in humans. Hum Mov Sci 14: 13–43.

[37] Adolph KE, Berger SE (2006) Motor Development. In: Handbook of child psychology: Vol 2: Cognition, perception, and language (6th ed.), edited by Damon W, Lerner R, Kuhn D and Siegler RS, New York: Wiley, 161–213.

[38] Malouin F, Richards CL (2000) Preparatory adjustments during gait initiation in 4–6-year-old children. Gait Posture 11: 239–253. 10802437 10.1016/s0966-6362(00)00051-5

[39] Campos JJ, Anderson DI, Barbu-Roth M, Hubbard EM, Hertenstein MJ, et al (2000) Travel broadens the mind. Infancy 1: 149–219. 10.1207/S15327078IN0102_1 32680291

[40] Isableu B, Ohlmann T, Crémieux J, Amblard B (2003) Differential approach to strategies of segmental stabilisation in postural control. Exp Brain Res 150: 208–221. 12677318 10.1007/s00221-003-1446-0

[41] Bertenthal BI, Boker S, Xu M (2000) Analysis of the perception-action cycle for visually induced postural sway in 9-month-old sitting infants. Infant Behav Dev 23: 299–316.

[42] Bertenthal BI, Rose JL, Bai DL (1997) Perception-action coupling in the development of visual control of posture. J Exp Psychol Hum Percept Perform 23: 1631–1643. 9425672 10.1037//0096-1523.23.6.1631

[43] Peterka RJ (2002) Sensorimotor integration in human postural control. J Neurophysiol 88: 1097–1118. 12205132 10.1152/jn.2002.88.3.1097

[44] Ornitz EM (1983) Normal and pathological maturation of vestibular function in the human child. In: Development of auditory and vestibular systems, edited by Romand R. New York, 479–536.

[45] Flanagan JR, Wing AM (1997) The role of internal models in motion planning and control: evidence from grip force adjustments during movements of hand-held loads. J Neurosci 17: 1519–1528. 9006993 10.1523/JNEUROSCI.17-04-01519.1997 PMC6793733

[46] Hore J, Watts S, Tweed D (1999) Prediction and compensation by an internal model for back forces during finger opening in an overarm throw. J Neurophysiol 82: 1187–1197. 10482738 10.1152/jn.1999.82.3.1187

[47] Adolph KE (1997) Learning in the development of infant locomotion. Monogr Soc Res Child 62: 1–158. 9394468

[48] Adolph KE (2000) Specificity of learning: Why infants fall over a veritable cliff. Psychol Sci 11: 290–295. 11273387 10.1111/1467-9280.00258

[49] Chen LC, Metcalfe JS, Jeka JJ, Clark JE (2007) Two steps forward and one back: Learning to walk affects infants’ sitting posture. Infant Behav Dev 30: 16–25. 17292776 10.1016/j.infbeh.2006.07.005

